# Characterization of thyroid metastasis from clear cell renal cell carcinoma on ultrasonography: a report of three cases and literature review

**DOI:** 10.1530/ETJ-23-0121

**Published:** 2023-12-22

**Authors:** Hai-Yan Jia, Juan Chen, Zi-Xin Zhai, Wen-Wen Fan, Si-Jie Yuan, Qiong Liu, Xiao-Hui Yan, Qian-Qian Shen, Li-Ping Liu

**Affiliations:** 1 Department of Ultrasound, First Hospital of Shanxi Medical University, Taiyuan, Shanxi, China; 2 Department of Ultrasound, Shanxi Provincial People's Hospital, Taiyuan, Shanxi, China

**Keywords:** thyroid, clear cell renal cell carcinoma, metastasis, ultrasonography

## Abstract

**Introduction:**

Thyroid metastasis from clear cell renal cell carcinoma (ccRCC) is relatively rare, so ultrasound doctors lack experience with the disease, which can easily lead to misdiagnosis. We describe three cases of thyroid metastasis from ccRCC detected 12, 8, and 7 years after nephrectomy.

**Case presentation:**

The first patient, a 78-year-old woman, was admitted to our institution for hoarseness and progressive dyspnea. Ultrasonography revealed bilateral thyroid nodules and abnormal cervical lymph nodes. Fine-needle aspiration biopsy (FNAB) and core needle biopsy (CNB) of the thyroid was nondiagnostic. The other two patients, a 54-year-old man and a 65-year-old man, were admitted to our institution for a goiter pressing on the trachea. In each case, ultrasonography revealed a partially cystic nodule of the left lobe of the thyroid gland. Histological examination of three patients after thyroidectomy showed thyroid metastasis from ccRCC.

**Discussion/Conclusion:**

For patients with a history of ccRCC, long-term follow-up and routine thyroid ultrasonography should be performed. If a new thyroid nodule is found during the examination, metastases should be highly suspected. FNAB should be performed, even if benign ultrasound features seem to be in evidence. If the diagnosis of FNAB is incorrect and inconclusive, CNB should be performed.

## Established facts

- Already known fact 1: Thyroid metastasis from ccRCC is relatively rare, so ultrasound doctors lack experience with the disease, which can easily lead to misdiagnosis. There is little discussion about its ultrasonographic characteristics and the important diagnostic role of FNAB/CNB in the previous literature.

## Novel insights

- New information 1: Thyroid metastasis from ccRCC mostly appeared as a single solid or partially cystic nodule with clear borders, usually without calcification, and with abundant blood flow signals.

- New information 2: For patients with a history of ccRCC, long-term follow-up and routine thyroid ultrasonography should be performed. If a new thyroid nodule is found during the examination, metastasis should be highly suspected. FNAB should be performed as early as possible to clarify the pathology. If the diagnosis of FNAB is incorrect and inconclusive, CNB should be performed.

Ultrasound is the most sensitive imaging method for the differential diagnosis of benign and malignant thyroid nodules. Thyroid metastasis from clear cell renal cell carcinoma (ccRCC) is relatively rare, with occult onset and no specific ultrasonographic characteristics, making it prone to misdiagnosis. In this study, we present three cases of thyroid metastasis from ccRCC to analyze their ultrasonographic characteristics and review the relevant literature. We aimed to improve the understanding of thyroid metastasis from ccRCC among ultrasound doctors and to provide a better diagnostic basis for clinical practice.

## Case reports

### Case 1

A 78-year-old female was admitted to our hospital in March 2021 due to hoarseness for 2 months and progressive dyspnea aggravated for 10 days. Physical examination revealed a tough nodule in the left thyroid gland approximately 5 cm in size, with unclear boundaries and moderate mobility, moving with deglutition. The medical history of the patient included left nephrectomy 12 years prior due to ccRCC and pancreaticoduodenectomy 3 years prior for pancreatic metastasis from ccRCC.

Thyroid function tests revealed that serum free triiodothyronine and serum free tetraiodothyronine were normal, serum thyrotropin was 9.69 µIU/mL (normal range: 0.27–4.2 µIU/mL), thyroglobulin antibody was 766.00 IU/mL (normal range: 0–115 IU/mL), and thyroid peroxidase antibody was 235.00 IU/mL (normal range: 0–34 IU/mL).

**Figure 1:**
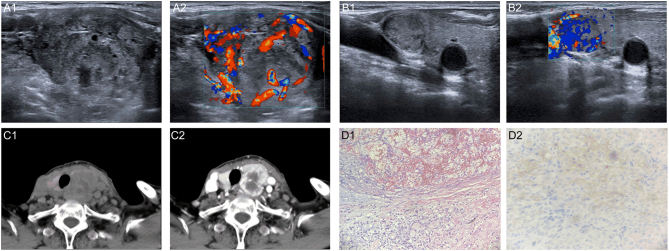
A 78-year-old woman with thyroid metastasis from ccRCC. A1. Transverse sonogram showing a heterogeneous hypoechoic nodule (4.2 × 3.3 × 2.9 cm) with irregular morphology in the left lobe. A2. Color Doppler sonogram shows abundant blood flow signals in the nodule. B1. Transverse sonogram showing a hypoechoic nodule (16 × 11 mm) with regular morphology in the right lobe. B2. Color Doppler sonogram image shows abundant blood flow signals in the nodule. C1. CT showing low-density masses in both lobes of the thyroid gland with poor demarcation from adjacent muscle and soft tissue. C2. Uneven enhancement on enhancement scan. D1. Surgical pathology confirming thyroid metastasis from ccRCC (HE ×200). D2. Immunohistochemistry shows CD10 (+) (HE ×400).

Ultrasonography revealed that the volume of the left lobe of the thyroid gland was increased, and a heterogeneous hypoechoic nodule was visible, measuring 4.2 × 3.3 × 2.9 cm, with irregular morphology, a clear boundary, and rich blood flow signals. A hypoechoic nodule could be seen in the right lobe, approximately 1.6 × 1.1 cm in size, with a clear boundary, regular morphology and abundant blood flow signals. The abnormal lymph nodes in left cervical areas III, IV, and VI were considered metastases. Based on their experience and the patient’s history, the ultrasound doctors classified the left lobe nodule as European Thyroid Imaging and Reporting Data System (EU-TIRADS) 5. The right lobe nodule was classified as EU-TIRADS 4 according to the conventional method. Computed tomography (CT) showed a malignant space-occupying lesion of the thyroid gland. Lymph node metastasis was diagnosed in the left cervical areas IV and V (shown in Fig. 1).

Fine needle aspiration biopsy (FNAB) and core needle biopsy (CNB) were performed on the left lobe nodule. The FNAB results were classified as Bethesda 2. CNB showed that the vast majority of samples were of degenerative necrotic and structurally indistinct tissues, with a few cytoplasmically translucent cells scattered throughout. Because of very limited sampling, no additional immunocytochemistry or immunohistochemistry examination was performed.

The patient underwent total thyroidectomy. Postoperative histopathological examination and immunohistochemical stains were compatible with thyroid metastasis from ccRCC (shown in Fig. 1). The left suprathyroidal vein was embolized. One metastatic lymph node in the left cervical areas III and IV was confirmed by pathology.

### Case 2

A 54-year-old male was admitted to our hospital in September 2022 due to a goiter pressing on the trachea. Physical examination showed that the thyroid gland was enlarged by a degree of II. A nodule measuring approximately 4.0 × 3.0 cm was palpable in the left thyroid gland, with clear borders and no pain on pressure, which moved with deglutition. The medical history of the patient included left nephrectomy 8 years prior due to ccRCC. Thyroid function tests were normal.

**Figure 2:**
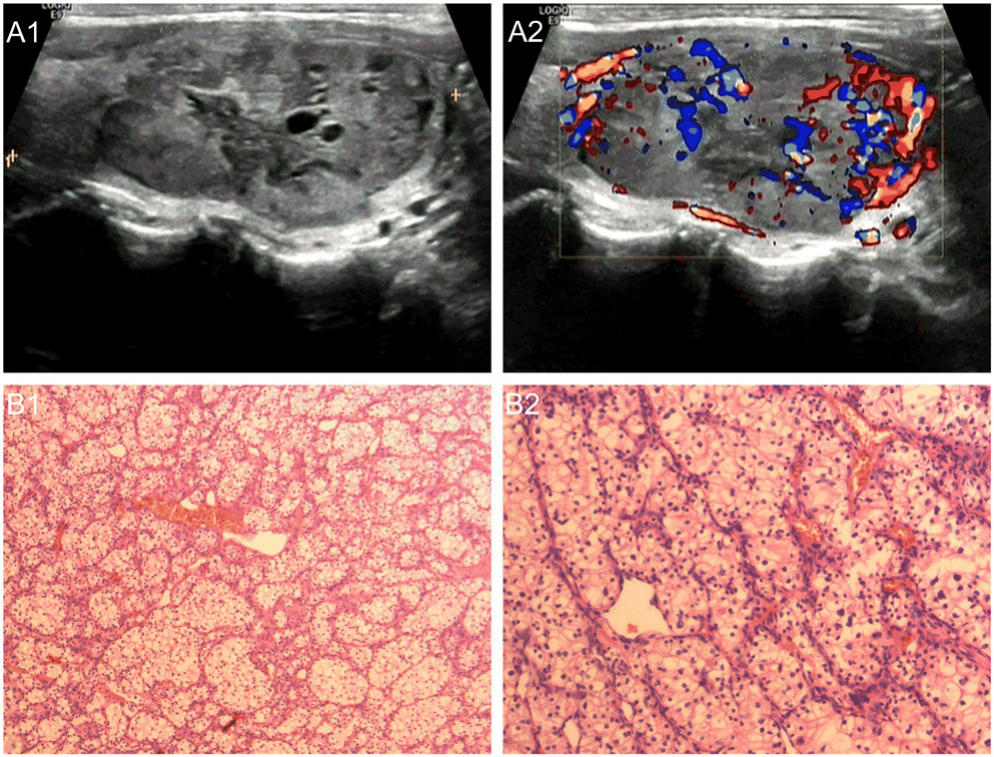
A 54-year-old man with thyroid metastasis from ccRCC. A1. Transverse sonogram showing a partially cystic nodule (4.5 × 2.0 × 3.5 cm) with clear borders and regular morphology in the left lobe. A2. Color Doppler sonogram image shows abundant blood flow signals in the nodule. B1 and B2. Hematoxylin and eosin staining; Surgical pathology confirming thyroid metastasis from ccRCC.

Ultrasonography revealed a partially cystic mass occupying the left lobe, measuring 4.5 × 2.0 × 3.5 cm, with regular morphology, clear boundaries and rich blood flow signals (shown in Fig. 2). The thyroid nodule was classified as EU-TIRADS 4 according to the conventional method. FNAB and CNB were not performed.

The patient underwent left thyroidectomy. Postoperative histopathological examination and immunohistochemical stains were compatible with thyroid metastasis from ccRCC (shown in Fig. 2).

Further postoperative ultrasound and enhanced CT showed multiple nodules in the pancreas, which were considered metastases.

### Case 3

A 65-year-old female was admitted to our hospital in May 2023 due to goiter compression on the trachea. Physical examination showed that the thyroid gland was enlarged by a degree of II. A nodule measuring approximately 5.0 × 3.0 cm that moved with deglutition was palpated in the left thyroid gland, with clear borders and no pain on pressure. The medical history of the patient included left nephrectomy seven years prior due to ccRCC. Thyroid function tests were normal.

**Figure 3:**
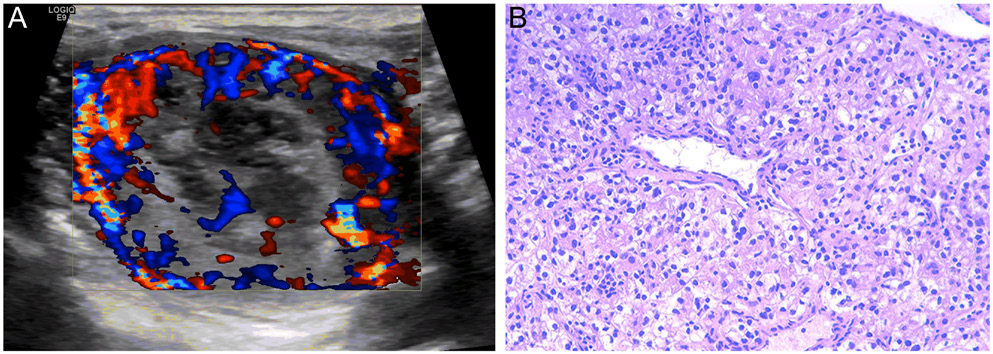
A 64-year-old woman with thyroid metastasis from ccRCC. A. Color Doppler sonogram shows abundant blood flow signals in the nodule. B. Hematoxylin and eosin staining; Surgical pathology confirming thyroid metastasis from ccRCC.

Ultrasonography revealed a partially cystic mass occupying the left lobe, measuring 4.5 × 3.3 cm, with regular morphology, clear boundaries and rich blood flow signals (shown in Fig. 3). The thyroid nodule was classified as EU-TIRADS 4 according to the conventional method. FNAB and CNB were not performed.

The patient underwent left thyroidectomy. Postoperative histopathological examination and immunohistochemical stains were compatible with thyroid metastasis from ccRCC (shown in Fig. 3).

## Discussion

Metastases in thyroid gland of other primary tumors are relatively rare, accounting for 1.4–3% of thyroid malignancies (1). Chung *et al.* (2) reported that among the tumors able to metastasize in thyroid gland, renal cell carcinoma is the most common. Yu *et al.* (3) found that only 5% of metastases in thyroid gland of other primary tumors in China were from the kidney. The reason for this difference may be related to the different incidence rates of each tumor in different countries. ccRCC is a highly aggressive tumor that most often metastasizes to the lung, bone and liver and less commonly metastasizes to the thyroid. Thyroid metastasis from ccRCC can present at the same time as the primary tumor is diagnosed (synchronous) or months to decades after the primary tumor diagnosis (metachronous) (2), which makes the diagnosis of thyroid metastasis from ccRCC more difficult. In this study, metastases were detected in three patients 12, 8, and 7 years after primary ccRCC surgery. Therefore, lifelong follow-up is recommended for patients with a history of ccRCC.

In this study, we retrospectively analyzed the ultrasound features of thyroid nodules in three patients with postoperative thyroid metastases from ccRCC (Table 1). We searched PubMed for articles published between 2012 and 2022 on thyroid metastasis from ccRCC, of which eight provided clear ultrasound images and summarized the ultrasound characteristics of the cases mentioned in the eight articles as shown in Table 2 (4, 5, 6, 7, 8, 9, 10, 11). The results showed that thyroid metastasis from ccRCC mostly appeared as a single solid or partially cystic nodule with clear borders, usually without calcification, and with abundant internal blood flow signals. The ultrasound characteristics of three patients in this study were similar to this result. The abundant blood flow signals is related to the biological characteristics of ccRCC, as its interstitium is rich in capillaries and blood sinuses, which are prone to hemorrhage (12). These features are different from the general ultrasound features of common primary malignant tumors of the thyroid gland. Thyroid metastasis from ccRCC tends to form nodular masses resembling primary thyroid tumors or even approximating the ultrasound features of benign nodules, with few or no suspicious malignant signs (13), which is one of the reasons why needle biopsies are often neglected by clinicians. Thyroid metastasis from ccRCC may be accompanied by cervical lymph node metastases and internal jugular vein tumor thrombus. Although the ultrasound features of thyroid metastases from ccRCC prove to be rather nonspecific, the presence of suspicious lymph nodes in patients with a history of ccRCC may raise suspicion of metastasis to a thyroid mass demonstrated on the same ultrasound examination, especially for thyroid masses without suspected malignant ultrasound characteristics.

**Table 1:** Ultrasonographic features of thyroid nodules in three patients with thyroid metastasis from clear cell renal cell carcinoma.

Case	Localization	Echo	Shape	Boundary	Calcification	Blood flow	Lymphatic metastasis
1	Left lobe	Hypoechoic	Irregular	Clear	Without	Rich	Yes
	Right lobe	Hypoechoic	Regular	Clear	Without	Rich	No
2	Left lobe	Partially cystic	Regular	Clear	Without	Rich	No
3	Left lobe	Partially cystic	Regular	Clear	Without	Rich	No

**Table 2:** Ultrasonographic features of thyroid metastasis from clear cell renal cell carcinoma reported in previous studies.

Study	Year	Cases reported, *n*	Ultrasonographic features
Vandemergel *et al.* (4)	2021	1	Single lesion, solid, hypoechoic, irregular shape, lobulated, without calcification, and with rich blood flow
Tian *et al.* (5)	2020	1	Multiple lesions, hypoechoic, solid-cystic, unclear boundary, with calcification, and with relatively rich blood flow
Al Abdrabalnabi *et al.* (6)	2019	1	Single lesion, solid-cystic, unclear boundary, without calcification
Song *et al.* (7)	2017	9	Single lesion (3/9), multiple lesions (6/9), solid (9/9), hypoechoic (9/9), clear boundary (8/9), with calcification (0/9), and with rich blood flow (9/9)
Gheorghiu *et al.* (8)	2016	1	Single lesion, mixed echoic, solid-cystic, clear boundary, without calcification and with rich blood flow in the solid region
Cilengir *et al.* (9)	2016	1	Single lesion, solid, hypoechoic, with clear boundary, irregular shape, lobulated, with microcalcification, and with rich blood flow
Kobayashi *et al.* (10)	2015	10	Single lesion (8/10), multiple lesions (2/10), solid (10/10), hypoechoic (10/10), clear boundary (10/10), irregular shape (9/10), without calcification (10/10), and with rich blood flow (10/10)
Andrioli *et al.* (11)	2014	1	Single lesion, solid, hypoechoic, without calcification, posterior irregular margins, and with rich blood flow

FNAB is the most effective and commonly used method for the preoperative evaluation of the benign status or malignancy of thyroid nodules due to its rapidity, safety, and economy. For cervical lymph nodes suspected of metastasis by ultrasound characteristics, FNAB should also be performed promptly to confirm the diagnosis. FNAB is not mandatory for thyroid nodules with benign US characteristics in clinical practice (14). When a new thyroid nodule develops in a patient with a history of ccRCC, a needle biopsy should be performed to clarify the pathology, even if the nodule has benign ultrasound features. A large series demonstrated that FNAB is a sensitive and specific method for detecting metastases in thyroid gland of other primary tumors (1). However, other reviews have shown that the false-negative rate of preoperative FNA diagnosis of metastases in thyroid gland of other primary tumors is as high as 26.3% (2). Recently, CNB has been effective for the diagnosis of thyroid nodules. CNB achieved better diagnostic performance than FNAB by obtaining larger tissue samples and additional histological information (7, 15, 16, 17, 18). For large and rapidly growing thyroid masses such as ATC or thyroid lymphoma or thyroid gland metastases from other tumors, CNB is the preferred and primary diagnostic tool (16, 17). However, CNB should be carefully performed, considering the more invasive nature and the location of several nerves and vascular structures around the thyroid gland. According to the current guidelines, CNB is recommended for thyroid nodules with repeated inadequate FNA cytology, repeated Bethesda class III cytology, and when histological assessment can improve preoperative diagnosis (e.g. suspicion of poorly differentiated or undifferentiated thyroid cancer, thyroid lymphoma, thyroid metastases) (19). For case 1, due to the abundant blood flow to the nodule, FNAB was performed after one CNB session, which failed to give a correct diagnosis. This may be related to the inadequate sampling, which does not allow for immunocytochemistry or immunohistochemistry examination.

Because clear cell components are present in both primary clear cell thyroid tumors and thyroid metastases from ccRCC, puncture and postoperative pathology are sometimes challenged in distinguishing primary cancer from metastasis, and immunohistochemistry may improve the diagnostic accuracy. ccRCC metastases are usually immunocytologically positive for RCC antigen, CD10 or vimentin and negative for thyroglobulin and thyroid transcription factor 1 (20).

For patients with a history of ccRCC, long-term follow-up and routine thyroid ultrasonography should be performed. If a new thyroid nodule is found during the examination, metastasis should be highly suspected, Even if the nodule does not have malignant ultrasound features, FNAB should be performed as early as possible to clarify the pathology. If the diagnosis of FNAB is incorrect and inconclusive, CNB should be performed. For large and rapidly growing thyroid masses, CNB should be performed directly.

## Declaration of interest

The authors declare that there is no conflict of interest that could be perceived as prejudicing the impartiality of this case report.

## Funding

This work was supported by Application Basic Research Project of Science and Technology Department of Shanxi Province (201801D121340), and Key Research and Developmenthttp://dx.doi.org/10.13039/100006190 Program of Science and Technology Department of Shanxi Province (201903D321190).

## Statement of ethics

This study involving human participants was approved by the Ethics Review Committee of First Hospital of Shanxi Medical University. The patients/participants provided written informed consent to participate in this study. Written informed consent was obtained from the individual(s) for the publication of any potentially identifiable images or data included in this article.

## Author contribution statement

H-YJ, JC, and Z-XZ wrote the manuscript and collected case data; L-PL designed the manuscript; W-WF, S-JY, QL, X-HY, and Q-QS revised the manuscript; all authors approved the version to be published.
